# Exploring BODIPY-Based Sensor for Imaging of Intracellular Microviscosity in Human Breast Cancer Cells

**DOI:** 10.3390/ijms23105687

**Published:** 2022-05-19

**Authors:** Dziugas Jurgutis, Greta Jarockyte, Vilius Poderys, Jelena Dodonova-Vaitkuniene, Sigitas Tumkevicius, Aurimas Vysniauskas, Ricardas Rotomskis, Vitalijus Karabanovas

**Affiliations:** 1 Biomedical Physics Laboratory, National Cancer Institute, P. Baublio St. 3b, 08406 Vilnius, Lithuania; dziugas.jurgutis@nvi.lt (D.J.); greta.jarockyte@nvi.lt (G.J.); vilius.poderys@nvi.lt (V.P.); ricardas.rotomskis@nvi.lt (R.R.); 2 State Research Institute Center for Physical Sciences and Technology, Sauletekio Ave. 3, 10257 Vilnius, Lithuania; aurimas.vysniauskas@ftmc.lt; 3 Institute of Chemistry, Faculty of Chemistry and Geosciences, Vilnius University, Naugarduko St. 24, 03225 Vilnius, Lithuania; jelena.dodonova@gmail.com (J.D.-V.); sigitas.tumkevicius@chf.vu.lt (S.T.); 4 Department of Chemistry and Bioengineering, Vilnius Gediminas Technical University, Sauletekio Ave. 11, 10223 Vilnius, Lithuania

**Keywords:** microviscosity, molecular rotors, fluorescence spectroscopy, live cancer cells, lipid droplets, fluorescence lifetime imaging

## Abstract

BODIPY-based molecular rotors are highly attractive imaging tools for imaging intracellular microviscosity in living cells. In our study, we investigated the ability to detect the microviscosity of biological objects by using BDP-NO_2_ and BDP-H molecular rotors. We describe in detail the optical properties of BDP-NO_2_ and BDP-H molecular rotors in aqueous media with and without proteins, together with their accumulation dynamics and localization in live and fixed human breast cancer cells. Furthermore, we investigate the applicability of these molecules to monitor microviscosity in the organelles of human breast cancer cells by fluorescence lifetime imaging microscopy (FLIM). We demonstrate that the BDP-NO_2_ molecular rotor aggregates in aqueous media and is incompatible with live cell imaging. The opposite effect is observed with BDP-H which preserves its stability in aqueous media, diffuses through the plasma membrane and accumulates in lipid droplets (LDs) and the cytosol of both live and fixed MCF-7 and MDA-MB-231 cancer cells. Finally, by utilizing BDP-H we demonstrate that LD microviscosity is significantly elevated in more malignant MDA-MB-231 human breast cancer cells, as compared to MCF-7 breast cancer cells. Our findings demonstrate that BDP-H is a water-compatible probe that can be successfully applied to measure microviscosity in the LDs of living cells.

## 1. Introduction

The last decade has witnessed huge growth in the research of microscopic viscosity (microviscosity) and its role in the intracellular micro-environment. Microviscosity is crucial in governing the structural and functional balance of subcellular components, regulating the diffusion of biomolecules through cells and significantly altering cellular behavior [1,2]. In addition, abnormal microviscosity alterations indicate cell death [3] or the onset of diseases, e.g., diabetes [4], atherosclerosis [5], Alzheimer’s disease [6] and cancer [7,8]. Monitoring microviscosity may enhance our current knowledge of these diseases at the cellular level and allow for earlier diagnosis.

The surge in microviscosity research would not have been possible without the emergence of viscosity-sensitive fluorophores termed ‘molecular rotors’. Intramolecular rotation of molecular rotors depends on the microviscosity of the surrounding environment: in a highly viscous cellular compartment (e.g., plasma membrane) the rotation is restrained, leading to a slower non-radiative relaxation from the excited state, thus resulting in a higher fluorescence intensity and longer fluorescence lifetime, and vice versa in a micro-environment with lower microviscosity (e.g., cytoplasm) [2,9,10,11,12]. Before application, molecular rotors are typically calibrated in organic solvent mixtures of known bulk viscosity. Thus, a calibration curve showing the fluorescence lifetime’s dependence on viscosity is obtained, which usually follows a modified form of the Förster-Hoffmann equation [2,10,13,14]. Next, fluorescence lifetimes of molecular rotors in the sample of interest are measured using time-correlated single photon-counting based fluorescence lifetime imaging microscopy (FLIM) [15,16]. The previously obtained calibration curve allows the calculation of viscosity values from the measured lifetimes and the creation so-called viscosity ‘maps’ [2].

**Figure 1:**
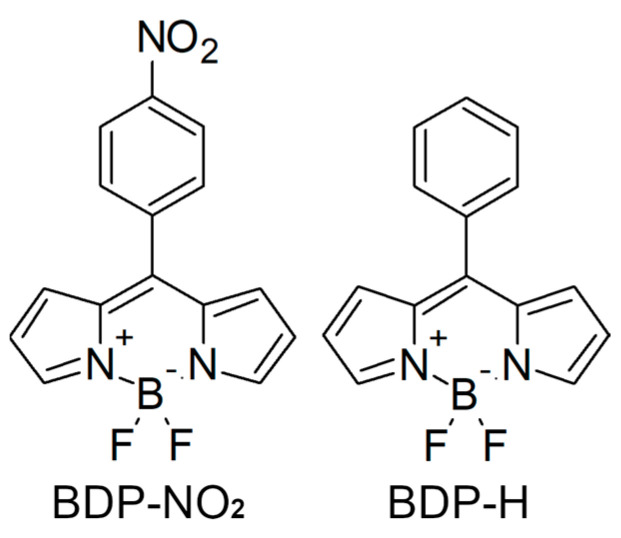
The molecular structures of BDP-NO_2_ and BDP-H molecular rotors examined in this work.

At present, boron-dipyrrin or BODIPY (4,4-difluoro-4-bora-3a,4a-diaza-s-indacene) based molecular rotors remain among the most widely used viscosity sensors since the first demonstration of such probes by Kuimova et al. [10]. A BODIPY core structure in molecular rotors grants large molar extinction coefficients, photostability and chemical inertness [17,18,19]. Furthermore, BODIPY-based probes can be easily functionalized at the appropriate positions to tune their sensitivity to micro-environment viscosity, polarity or temperature; increase the emission wavelength or Stokes shift; or direct the molecules to specific cells or organelles [20,21,22,23,24,25]. Recently, we demonstrated that the functionalization of *meso*-phenyl BODIPY (BDP-H) (Figure 1) with a heavy-electron withdrawing –NO_2_ group results in a viscosity-sensor with an extremely wide viscosity-sensitivity range (0.5–50,000 cP) [21]. Originally, a BDP-H molecule was examined as an accessory pigment for a BODIPY-porphyrin light-harvesting array [26]. Meanwhile, an initial investigation of BDP-NO_2_ (Figure 1) only determined its spectroscopic properties in acetonitrile [27]. Both BDP-NO_2_ and BDP-H were already tested in organic solvents of different viscosity and polarity and their sensitivity to viscosity was proven [21]. While organic solvents may prove useful for exploring sensitivity to polarity and temperature, as well as the underlying working mechanism of molecular rotors, they do not represent the conditions found within biological models. The variety of biomolecules and the complexity of the subcellular microenvironment may result in unforeseen interactions. This is enough to disturb the rotational mechanism of the molecular rotor and inhibit its sensitivity to microviscosity [28]. For this reason, in this study we conduct steady-state and time-resolved spectroscopies of both BDP-NO_2_ and BDP-H in phosphate-buffered saline (PBS) with or without fetal bovine serum (FBS).

Our study seeks to determine if BDP-NO_2_ and BDP-H molecular rotors are applicable for monitoring the microviscosity of biological samples, as both molecules were not tested in cells [12,21,29]. While there has been an abundance of research on the microviscosity of HeLa human cervical cancer cells and SK-OV-3 human ovarian cancer cells [10,30,31,32,33], the intracellular microviscosity of human breast cancer cells remains poorly understood. There are several subtypes of breast cancers based on their expression of progesterone receptors (PR) and oestrogen receptors (OR), as well as the expression of human epidermal growth factor receptor-2 (HER2) [34]. Cancer cells of these subtypes exhibit a plethora of genotypic and phenotypic differences [35,36]. For example, MCF-7 human breast cancer cells express PR, OR, and are of luminal A subtype, thus the cells are sensitive to hormone-therapy and are characterized as non-invasive cells with low metastatic potential [37]. Another example is MDA-MB-231 cells, which are triple-negative (PR-, OR- and HER-) and of basal subtype. These cells are more mesenchymal, highly-aggressive and metastatic [38,39]. Microviscosity could serve as a hallmark for identifying the different subtypes of breast cancer cells, or it may even enable the identification of therapy-resistant tumor cells or cancer stem cells based on their microviscosity. Therefore, the accumulation and viscosity sensing properties were investigated in two different human breast cancer cell lines, OR and PR positive MCF-7 and the more aggressive triple-negative MDA-MB-231, with the aim to determine the optimal incubation conditions, and to quantitatively compare microviscosity within the organelles of these cells.

## 2. Results and Discussion

### 2.1. Photophysical Characterization

**Figure 2:**
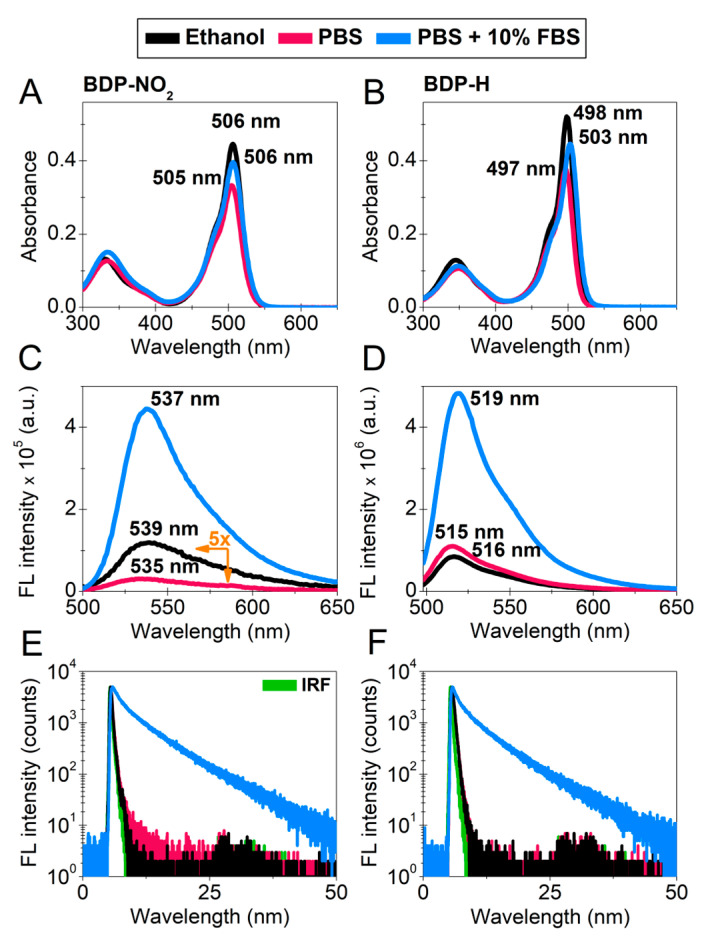
Photophysical characterization of BDP-NO_2_ and BDP-H diluted in ethanol (black), PBS (red) or PBS + 10% FBS (blue). (**A**,**B**) absorption spectra. (**C**,**D**) fluorescence (FL) spectra under the same quantity of the absorbed light quanta, λ_ex_ = 488 nm. (**E**,**F**) comparison of time-resolved FL decays, λ_ex_ = 473 nm. IRF—instrument response function (green). Normalized absorption and FL spectra of both molecular rotors are displayed in Supplementary Materials Figure S1. Fitting parameters of the FL decays are displayed in Supplementary Materials Table S1.

The spectral properties of 9 µM BDP-NO_2_ molecular rotor were evaluated in ethanol and 7.4 pH phosphate-buffered saline (PBS). BDP-NO_2_ absorption measurements reveal two absorbance bands: the peak of the main band in ethanol is at 506 nm, while a higher-energy band has a peak of 330 nm (Figure 2A). The fluorescence spectra of BDP-NO_2_ in ethanol reveal a band at 500–700 nm, with the maximum fluorescence at 539 nm (Figure 2C).

This concurs well with the previous findings that were obtained in our study [21]. The spectral measurements of BDP-NO_2_ in PBS resulted in absorbance and fluorescence spectra with the maxima of 505 nm and 535 nm, respectively (Figure 2A,C). Interestingly, dilution of BDP-NO_2_ in PBS resulted in a decrease in both the absorption and fluorescence intensity. This decrease could be explained by the lower solubility of BDP-NO_2_ in aqueous media or the formation of aggregate species. In addition, the intensity of the observed absorption bands dwindled with time (65.23% decrease in 30 min) (Supplementary Materials Figure S2A), thus indicating that due to interaction with PBS optical properties of the molecule are not stable. To elucidate this, we compared the fluorescence decays of the molecule in ethanol and in PBS (Figure 2E). Results revealed that in ethanol, BDP-NO_2_ fluorescence decay is monoexponential (64 ps). In contrast, measuring BDP-NO_2_ immediately after diluting it in PBS results in biexponential fluorescence decay and the average lifetime grows to 131 ps (Supplementary Materials Table S1). It is known that aggregates of the unmodified form of BODIPY molecules exhibit additional fluorescence band, and biexponential fluorescence decays are also produced [29]. This further reinforces the presence of aggregates in our solution.

Additionally, we investigated how fetal bovine serum (FBS) affects the optical properties of BDP-NO_2_. FBS is one of the components in cell growth medium and contains a mixture of biomolecules: proteins, amino acids, lipids, sugars, etc. [40]. Upon dilution of BDP-NO_2_ in PBS with 10% FBS, we observed a decrease in the main absorption band of BDP-NO_2_ at 506 nm (Figure 2A). As opposed to absorbance, BDP-NO_2_ fluorescence intensity increased significantly with the addition of FBS molecules. This is possibly due to the increased viscosity of the medium or BDP-NO_2_ localization within hydrophobic regions of FBS proteins and a smaller degree of aggregation. However, the decrease in BDP-NO_2_ absorbance with time was still observed, although at a much slower rate (14.58% decrease in 30 min) (Supplementary Materials Figure S2B). Therefore, the optical stability of BDP-NO_2_ remains impeded in aqueous media with or without FBS.

Another BODIPY based molecular rotor—BDP-H was also tested. The spectral characteristics of 9 µM BDP-H in PBS buffer differed little from those obtained in ethanol: the peaks of the absorption spectra were 497 nm and 498 nm, while the peaks of the fluorescence spectra were 515 nm and 516 nm, respectively (Figure 2B,D). In addition, the fluorescence intensity of BDP-H in PBS was one order of magnitude larger, as compared to BDP-NO_2_ in PBS at the same measuring conditions (Figure 2C,D). Moreover, BDP-H diluted in PBS is relatively photostable (Supplementary Materials Figure S3). Analysis of the time-resolved fluorescence decays (Figure 2F) revealed that BDP-H dilution in PBS or ethanol results in monoexponential fluorescence decays with similar fluorescence lifetimes: 264 ps and 261 ps, respectively (Supplementary Materials Table S1). The differences in the spectral properties of BDP-H in aqueous solutions and organic solvents are minimal, confirming that the molecular rotor is soluble in water [29].

Upon dilution of BDP-H in PBS with 10% FBS we observed a red-shift of 6 nm and a decrease (8.2%) in the main absorption band of BDP-H at 497 nm (Figure 2B). The inclusion of FBS in PBS results in a bathochromic shift of BDP-H fluorescence maximum by 4 nm and a significant increase in fluorescence intensity (Figure 2D). In contrast to BDP-H diluted with PBS, the addition of serum proteins results in multiexponential fluorescence decay and *τ*_Av_ increases to 4850 ps (Figure 2F). Typically, the multiexponential decays of molecular rotors are associated with the localization of these molecules in distinct environments with a different microviscosity [11], aggregation [8,41], or interaction with certain biomolecules such as amino acids or proteins [42]. As BDP-H is compatible with aqueous media and we observe no aggregation, the only plausible explanation of the observed spectral changes is BDP-H interaction with the components of FBS.

To elucidate the nature of BDP-H multiexponential fluorescence decays in PBS with 10% FBS, we conducted an additional analysis of BDP-H diluted in PBS with increasing concentrations of bovine serum albumin (BSA)—the most abundant component of FBS (Supplementary Materials Figure S4). Titration of BDP-H with BSA results in a gradual red-shift of both absorption and fluorescence spectra (Supplementary Materials Figure S4A,C) until the ratio reaches 1:1 (Supplementary Materials Figure S4B). Likewise, we also observe the growth of fluorescence lifetimes with the increasing concentration of BSA (Supplementary Materials Figure S4D). The findings unveil that the observed red-shift in the spectral properties of BDP-H is a result of interaction between BDP-H and BSA molecules. Presumably, the BDP-H molecule enters the hydrophobic pocket of the protein where the rotation of the molecular rotor is restricted, imitating a higher microviscosity environment, and resulting in increased fluorescence intensity and longer lifetimes. However, this interaction can be completely altered in the cellular environment. Thus, it is paramount to test molecular rotors both in aqueous media (with or without FBS molecules) and in living cells to identify whether the properties, localization and fluorescence lifetime of the microviscosity-sensor is altered in the presence of proteins.

### 2.2. Accumulation of BDP-NO_2_ and BDP-H in Breast Cancer Cells

**Figure 3:**
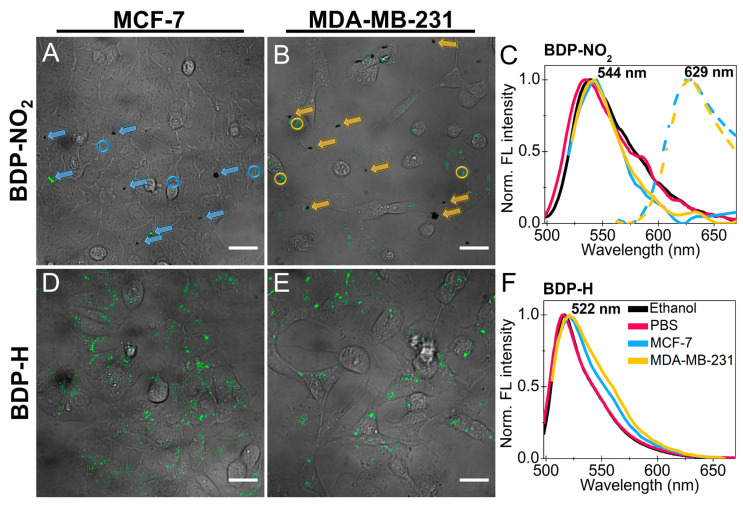
Comparison of BDP-NO_2_ and BDP-H staining (**A**,**B**,**D**,**E**) and fluorescence (FL) spectra (**C**,**F**) in live MCF-7 and MDA-MB-231 cancer cells. (**A**,**B**) brightfield images merged with fluorescence (FL) images of cancer cells stained with BDP-NO_2_ diluted in PBS; scale bars: 30 µm. Blue and yellow arrows mark BDP-NO_2_ aggregates and circles denote the spots within which FL spectra of BDP-NO_2_ were measured with a 32-channel spectral detector. (**C**) comparison of BDP-NO_2_ diluted in ethanol (black) or PBS (red) FL spectra and FL spectra of the molecular rotor from live MCF-7 (blue) and MDA-MB-231 (yellow) cells (all spectra in solid lines). Dashed lines were obtained from BDP-NO_2_ aggregates marked with arrows in A and B; λ_ex_ = 480 ± 5 nm. (**D**,**E**) brightfield images merged with FL images of cancer cells stained with BDP-H diluted in PBS; scale bars: 30 µm. (**F**) comparison of BDP-H diluted in ethanol (black) or PBS (red) FL spectra and FL spectra of the molecular rotor from live MCF-7 (blue) and MDA-MB-231 (yellow) cells; λ_ex_ = 480 ± 5 nm. Blue and yellow FL spectra in F were obtained by averaging FL spectra from 10 different spots within cells, where BDP-H is accumulated, and subtracting the background FL spectra, averaged from 5 different points.

Even though the BDP-NO_2_ molecular rotor displays poor solubility in aqueous media, we decided to determine whether its accumulation in living cells is also impeded. Our experiments demonstrate that incubation with the microviscosity-sensor diluted in PBS results in visible aggregates forming around the cells (Figure 3A,B, colored arrows). Application of a 32-channel spectral detector revealed that the aggregates are characterized with a fluorescence maximum of 629 nm (Figure 3C, dashed lines). Even with the aggregates, the interior of both MCF-7 and MDA-MB-231 cells still exhibit minimal staining with a fluorescence spectra maximum at 544 nm (Figure 3C, solid lines). A similar fluorescence spectra maximum (539 nm) was observed when BDP-NO_2_ was diluted in ethanol. Thus, part of the BDP-NO_2_ molecules remain non-aggregated and successfully internalize into live cancer cells. Regardless, our findings prove that the molecule by itself cannot be subjected to microviscosity monitoring in live biological models. Nonetheless, the encapsulation of BDP-NO_2_ in liposomes could prevent its aggregation and would assure the effective delivery of the molecule within the intracellular environment. Such delivery of a *β*-phenyl substituted BODIPY molecular rotor was already demonstrated [43].

In contrast to BDP-NO_2_, the BDP-H photophysical properties in aqueous media remain relatively unchanged. Consequently, testing the molecular rotor in living cells revealed a higher accumulation of BDP-H, as compared to BDP-NO_2_ (Figure 3). BDP-H acquires punctate-like distribution in membrane-bound organelles, as seen in both the MCF-7 and MDA-MB-231 cancer cell images, and no aggregates are observed outside the cell structure (Figure 3D,E). In case of dye aggregation, an additional fluorescence band in the red region (>630 nm) would be observed which is characteristic of the aggregates of the unmodified BODIPY molecule [29]. Since no additional fluorescence bands are observed in the fluorescence spectra of BDP-H from vesicular structures and the form of the fluorescence spectra is similar as the one registered in ethanol or PBS (Figure 3F), we conclude that BDP-H does not form aggregates in cells.

Furthermore, to determine the entry process of BDP-H we compared the accumulation of the molecular rotor in live and fixed cancer cells (Supplementary Materials Figure S5). In this work, a 4% paraformaldehyde (PFA) solution was applied for fixation which is more suitable for preserving cellular lipids and the composition of LDs [44]. Experiments highlight that the BDP-H molecular rotor stains both fixed and live cells with a similar pattern: punctate-like dye distribution with a slight cytosolic staining. It is known that fixation completely stops all active cellular processes, including active transport. Thus, an unaltered dye localization in PFA fixed cells implies that the BDP-H molecular rotor passes the cell membrane via diffusion.

**Figure 4:**
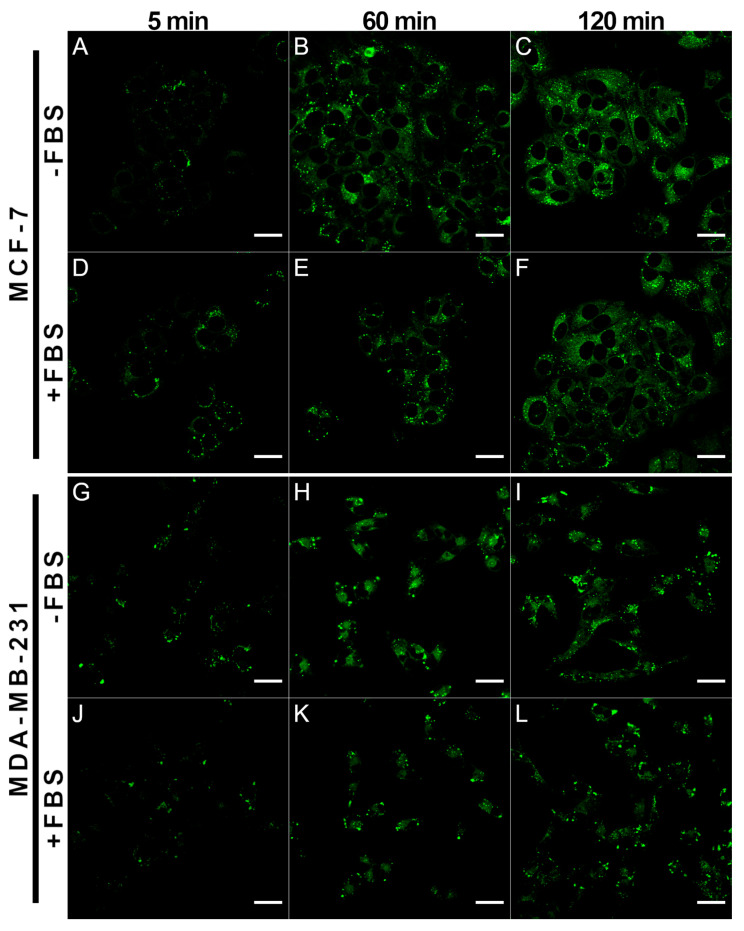
Intracellular distribution of 9 µM BDP-H in live MCF-7 (**A**–**F**) and MDA-MB-231 (**G**–**L**) cancer cells. Both cell lines were incubated in PBS without FBS (-FBS) or with 10% FBS (+FBS). Different incubation times with BDP-H (5–120 min) were used. Cells were visualized using laser-scanning confocal microscope; λ_ex_ = 480 ± 5 nm. Scale bars: 30 µm.

Next, we tested how the incubation time (5–120 min) affects the accumulation of the BDP-H molecular rotor in live cancer cells (Figure 4). Even after 5 min, the dye was already distributed in a cytosol of MCF-7, providing additional evidence that BDP-H stains the interior of cells via diffusion (Figure 4A). Incubation of cells with BDP-H for 60 min resulted in sufficient staining of the intracellular structures (Figure 4B,H). A longer incubation duration, e.g., 120 min, resulted in a negligible increase in FL intensity (Figure 4C,I). Thus, we conclude that the optimal incubation time with a BDP-H molecular rotor is 60 min.

As supplementation with FBS could affect the intracellular distribution of BDP-H, we also conducted experiments with both cancer cell lines treated with BDP-H diluted in PBS without FBS (Figure 4A–C,G–I) or in PBS containing 10% FBS (Figure 4D–F,J–L). Our findings indicate that despite the interaction between protein molecules and BDP-H, the fluorophore enters the cell and accumulates in both the cytosol and membrane-bound organelles in a similar fashion. This implies that the interaction between BDP-H and FBS proteins is not strong enough to prevent the diffusion of the molecular rotor through the plasma membrane. If BDP-H accumulation would be altered with the addition of FBS, this would indicate that BDP-H remains “attached” to protein molecules, making the molecule unsuitable for monitoring the microviscosity of intracellular organelles. To sum up, our findings indicate that despite BDP-H interaction with FBS proteins, the molecular rotor is applicable for measuring microviscosity in live cancer cells.

### 2.3. Localization of BDP-H in Breast Cancer Cells

**Figure 5:**
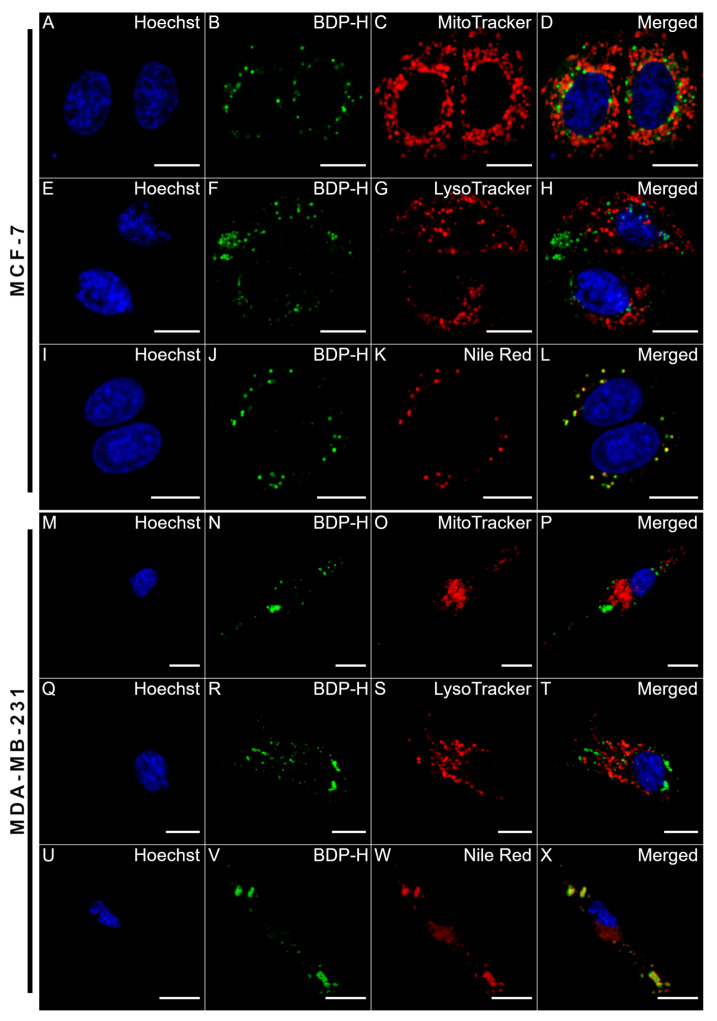
Confocal images of live human breast cancer cells treated with 37.5 µM Hoechst nuclei stain (λ_ex_ = 410 ± 5 nm) (**A**,**E**,**I**,**M**,**Q**,**U**), 9 µM BDP-H (λ_ex_ = 480 ± 5 nm) (**B**,**F**,**J**,**N**,**R**,**V**) and red-emitting commercial organelle probes: 200 nM MitoTracker Red FM, 75 nM LysoTracker Deep Red or 314 nM Nile Red (λ_ex_ = 560 ± 5 nm) (**C**,**G**,**K**,**O**,**S**,**W**). (**D**,**H**,**L**,**P**,**T**,**X**) merged images of Hoechst, BDP-H and one of the three red-emitting commercial probes. Scale bars: 15 µm. Results from colocalization analysis are shown in Supplementary Materials Figure S6.

For utilizing BDP-H as a microviscosity sensor, it was necessary to determine its localization within the cell. We hypothesized that the punctate-like distribution of BDP-H could be attributed to the dye accumulated in the mitochondria, lysosomes, or LDs. Therefore, we utilized red-emitting commercial probes (MitoTracker Red FM, LysoTracker Deep Red and Nile Red) to label each organelle type (Figure 5). Merged images revealed that there is no overlap between BDP-H and MitoTracked Red FM dye in both cell lines (Figure 5D,P). This was confirmed by relatively low Pearson correlation coefficients (PCC) that were obtained from the colocalization analysis in MCF-7 (PCC = 0.19 ± 0.02) and MDA-MB-231 (PCC = 0.10 ± 0.07) cells (Supplementary Materials Figure S6). In addition, BDP-H does not stain lysosomes (Figure 5H,T), as the PCC values of BDP-H and Lysotracker Deep Red colocalization were extremely low: 0.07 ± 0.05 and 0.11 ± 0.06 in MCF-7 and MDA-MB-231 cells, respectively (Supplementary Materials Figure S6). In contrast, the labelling of LDs with Nile Red revealed a complete overlap with the BDP-H molecular rotor in both MCF-7 and MDA-MB-231 cancer cells (Figure 5L,X) and relatively high PCC values were observed: 0.65 ± 0.06 and 0.79 ± 0.08 (Supplementary Materials Figure S6). This concurs well with previous findings of the BODIPY-C_12_ molecular rotor which exhibits a similar staining pattern and accumulates in vesicular structures, that were later identified as LDs [10,30,45]. Therefore, our results offer compelling evidence that the dot-like staining of vesicular structures is caused by an accumulation of BDP-H in the LDs of cancer cells.

LDs are organelles that are formed in the endoplasmic reticulum and are essential for lipid metabolism, trafficking and storage, membrane component synthesis, and for preventing lipotoxicity [46,47,48]. The interior of these organelles consists of densely arranged triacylglycerols and cholesterol esters (CE) that are covered by a monolayer of amphipathic lipids (mainly phospholipids) with surface proteins such as the perilipin family proteins [49,50,51]. Studies show that LDs, after their formation, form a connection with the membrane of the endoplasmic reticulum, through which a reciprocal exchange of lipids and surface proteins takes place [52,53]. Since the endoplasmic reticulum is in the vicinity of the nucleus, the dot-like staining of LDs is observed predominantly around the nuclei of cells, as confirmed by our results in both MCF-7 and MDA-MB-231 cancer cells (Figure 4 and Figure 5B,F,J,N,R,V).

The reasons for the weak cytosolic staining that is also observed are unclear. Autofluorescence of cells can be ruled out since imaging parameters were chosen specifically to nullify it. A possible explanation is that cytosolic staining is observed due to the BDP-H interaction with proteins in the cytosol of cells. Within cells, the amount and diversity of proteins and other biomolecules is far higher than in PBS with 10% FBS. Within the MCF-7 cell alone, the protein mass is about 100 pg, and the number of different protein groups can reach up to 3700 [54]. As a result, it is plausible that the majority of BDP-H accumulates in LDs with only a small amount remaining within the cytosol due to interactions with protein molecules.

### 2.4. Utilizing BDP-H and FLIM to Measure Lipid Droplet Microviscosity in Cancer Cells

**Figure 6:**
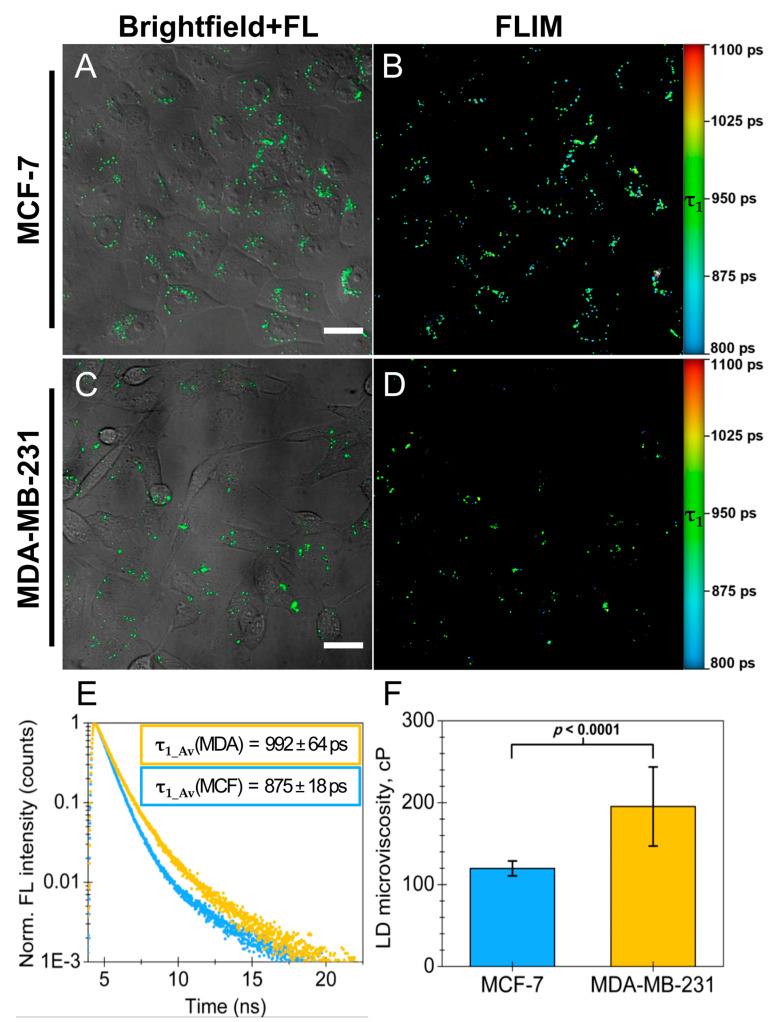
FLIM based comparison of lipid droplet (LD) microviscosity in live MCF-7 (**A**,**B**) and MDA-MB-231 (**C**,**D**) cells. (**A**,**C**) brightfield images merged with fluorescence (FL) images of cancer cells stained with BDP-H diluted in PBS. Scale bars: 30 µm; λ_ex_ = 480 ± 5 nm. (**B**,**D**) FLIM images, where the pixel colors indicate the value of FL decay time component *τ*_1_. (**E**) averaged FL decays obtained from biexponential fitting of 40 (20 for each cell line) FLIM region of interest (ROI) images containing only the LDs of single MCF-7 cells (MCF: blue decay) or single MDA-MB-231 cells (MDA: yellow decay). Mean ± SD values of *τ*_1_ component obtained from fitting are displayed in rectangles. (**F**) comparison of microviscosity values in LDs of MCF-7 and MDA-MB-231 cells. Bars show mean ± SD (*n* = 20 cells for each cell line). Microviscosity differences were statistically significant (Welch’s two-samples *t*-test, *t*(20.34) = 6.88; *p* < 0.0001). For FLIM images, the fluorescence was excited at 480 ± 5 nm and detected through a 578/105 nm filter. Fitting results of the FLIM images (**B**,**D**) are displayed in Supplementary Materials Figures S10 and S11, and Table S4.

Next, we assessed the utility of BDP-H in measuring the microviscosity of LDs in live cell cultures. FLIM measurements of live MCF-7 and MDA-MB-231 cells that were stained with BDP-H revealed monoexponential fluorescence decays in both cancer cell lines. While biexponential fits were required to fit the FLIM images, the emergence of a second exponential component (*τ*_2_) in fluorescence decays is observed due to the autofluorescence of cells. FLIM images of both MCF-7 and MDA-MB-231 control samples confirmed an autofluorescence signal (Supplementary Materials Figures S7 and S8, and Table S2). For these reasons, we present FLIM images containing only the shorter fluorescence decay component—*τ*_1_, obtained by a biexponential pixelwise fitting of LDs in the whole image (Figure 6B,D). The shorter component represents the fluorescence lifetimes of BDP-H molecules residing in LDs and can be applied to estimate LD microviscosity. For this purpose, we chose an BDP-H calibration curve obtained at 40 °C in toluene-castor oil mixtures of varying viscosity (Supplementary Materials Figure S9). Non-polar toluene-castor oil mixtures better reflect the core composition of LDs as compared to polar methanol-glycerol mixtures. It is crucial to take this into account when utilizing BDP-H because the molecular rotor exhibits slight sensitivity to solvent polarity [21]. Microviscosity values of 120 ± 9 cP and 195 ± 48 cP were assigned to the LDs in MCF-7 and MDA-MB-231 cells, respectively (Figure 6E,F and Supplementary Materials Table S3). The values concur well with previous findings in the literature [10,30,43]. For example, the first investigations into the application of the BODIPY-C_12_ molecular rotor by Kuimova et al. and Levitt et al. determined that the microviscosities of LDs in SK-OV-3 human ovarian cancer cells are 140 ± 40 cP and 160 ± 20 cP, respectively [10,30]. In latter studies, mixtures of methanol-glycerol were used to obtain the calibration curves. A recent study by Maleckaite et al. demonstrated that the LDs of live MCF-7 cells exhibit a microviscosity of 123 cP (calibration curve obtained in toluene-castor oil mixtures) [43]. Interestingly, previous research into the LDs of healthy cells, e.g., adipocytes and Chinese hamster ovary cells, observed markedly lower microviscosity values of ca. 57 cP and 34 ± 3 cP, respectively [45,55]. Perhaps cancer cells exhibit an elevated LD microviscosity as compared to healthy cells due to impaired lipid metabolism, de novo lipid synthesis, or altered lipid composition [56,57]. However, further research is obligatory to confirm this assumption.

The most interesting result to emerge from the data is the difference between the LD microviscosity in MCF-7 (120 ± 9 cP, *n* = 20) and MDA-MB-231 cells (195 ± 48 cP, *n* = 20) which is statistically significant (Welch’s two-samples *t*-test, *t*(20.34) = 6.88, *p* < 0.0001) (Figure 6F). To our knowledge, this is the first example in the literature where LD microviscosity is compared between cancer cells of different malignancy. The observed microviscosity differences reflect the distinct lipid phenotypes of LDs in MCF-7 and MDA-MB-231 cancer cells. While the core of most LDs mainly consists of neutral lipids, i.e., CE and triacylglycerols, their amount may vary between different cells [51]. Particularly, the amount of CE in LDs plays a pivotal role in the cellular mechanisms linked to the malignancy and proliferation of breast cancer [57,58,59,60]. Triple-negative MDA-MB-231 cells store higher amount of triacylglycerols and CE in their LDs in comparison to MCF-7 cells [61]. Aberrant accumulation of CE is also a characteristic of metastatic prostate cancer [62]. Given that cholesterol content regulates both the fluidity and microviscosity of membranes [63,64], it is expected that higher amounts of CE in LDs will lead to greater microviscosity. The aforementioned findings collectively support the hypothesis that an elevated LD microviscosity in MDA-MB-231 breast cancer cells is a consequence of an increased amount of CE in LDs.

Furthermore, the composition of LDs is linked to the stemness of cancer cells. For instance, ovarian cancer stem cells, instead of taking lipids from the outside, begin to actively perform de novo lipid synthesis, leading to elevated amounts of unsaturated fatty acids within LDs [56]. Application of BDP-H in cancer cells with varying stemness could hypothetically allow evaluation of the differences of LDs, or even enable the identification of cancer stem cells based on the microviscosity of their LDs.

## 3. Material and Methods

### 3.1. Molecular Rotors

BDP-NO_2_ and BDP-H molecular rotors (Figure 1) were synthesized as described previously [21]. 9 mM stock solutions of both molecular rotors were made in DMSO (Sigma-Aldrich, Taufkirchen, Germany). For the experiments, stock solutions were further diluted (1:1000) in ethanol or 7.4 pH phosphate-buffered saline (PBS) with or without 10% (*v*/*v*) fetal bovine serum (FBS) (all from Gibco, Waltham, MA, USA). The final concentration of both dyes was 9 µM (0.1% DMSO).

### 3.2. Commercial Probes Used for Colocalization Experiments

To label the mitochondria of live breast cancer cells, we applied MitoTracker Red FM (Invitrogen, Eugene, OR, USA). The stock solution was obtained by dissolving the lyophilized product in DMSO to a concentration of 1 mM. The final working concentration of 200 nM was obtained by diluting the stock solution in Dulbecco’s phosphate-buffered saline containing Ca^2+^ and Mg^2+^, w/o Phenol Red (DPBS) (Cegrogren Biotech, Ebsdorfergrund, Germany). For lysosome staining, we applied LysoTracker Deep Red (Invitrogen). The stock solution was diluted in Dulbecco’s Modified Eagle Medium (DMEM) w/o Phenol Red (Gibco) to a final working concentration of 75 nM. To label LDs, we utilized Nile Red (Invitrogen). 1 mg/mL Nile Red stock solution in DMSO was diluted in DPBS to a final working concentration of 314 nM. Finally, 37.5 µM Hoechst 33258 (Sigma-Aldrich) was applied for staining the nuclei of live cancer cells.

### 3.3. Spectral Analysis

Steady-state absorption spectra were recorded with a Cary 50 UV-VIS spectrophotometer (Varian Inc., Mulgrave, Australia). Fluorescence spectra were recorded with a Cary Eclipse spectrophotometer (Varian Inc.). Fluorescence decay measurements were conducted with a FL920 spectrophotometer (Edinburgh Instruments, Livingston, UK) and F900 software package. Fluorescence decays had 5000 counts at the peak of the decay. During the measurements, the temperature was kept constant at 25 °C. For all measurements, quartz cuvettes with an optical path length of 10 mm were applied.

### 3.4. Analysis of Spectral Measurements

Fluorescence decays of BDP-H and BDP-NO_2_ were fitted using the FAST (Fluorescence Analysis Software Technology) software package (v.3.1, Edinburgh Instruments, Livingston, UK). To obtain the fitting parameters, discrete component analysis with reconvolution was applied. To measure the instrument response function (IRF), polymer microspheres (size = 92 ± 3 nm) in aqueous suspension 3000 Series Nanosphere Size Standards (Thermo Fisher Scientific, Fremont, CA, USA) were utilized. Average fluorescence lifetimes were calculated using the following formula:

(1) $$\tau_{\mathrm{Av}}=\frac{\sum_{i}a_{i}\tau_{i}^{2}}{\sum_{i}a_{i}\tau_{i}}$$

where *i*—the number of the component: *a_i_*—amplitude of the *τ_i_* component; *τ_i_*—fluorescence lifetime of the *i* component; *τ*_Av_—the mean fluorescence lifetime.

### 3.5. Cell Culturing

For the cell experiments, two human breast cancer cell lines were used, namely MCF-7 and MDA-MB-231. The MCF-7 cell line was purchased from the European Collection of Cell Cultures and the MDA-MB-231 cell line was purchased from the American Type Culture Collection. Cells were cultured in DMEM, supplemented with 10% FBS, 100 U/mL penicillin and 100 µg/mL streptomycin (all from Gibco). The cells were maintained in a 37 °C incubator (Binder, Tuttlingen, Germany) with a humidified atmosphere containing 5% CO_2_. The cells were routinely subcultured 2–3 times a week in 25 cm^2^ culture dishes.

### 3.6. Treatment of Cells with Molecular Rotors and Commercial Probes

For intracellular imaging studies, the cells were seeded into an 8-chambered cover glass plate (Nunc, Lab-Tek) with a density of 3 × 10^4^ cells/chamber and subsequently incubated at 37 °C in a humidified atmosphere containing 5% of CO_2_ for 24 h. For the BDP-H accumulation dynamics and intracellular localization evaluation in live cells, cells were treated with 9 µM BDP-H and incubated for the next 5, 60 or 120 min. For fixed cells BDP-H staining, the cells were fixed by treating them for 15 min with 4% paraformaldehyde (PFA) (Sigma-Aldrich, St. Louis, MO, USA). Then, the cells were washed again with PBS three times and stained with BDP-H for 60 min.

For the colocalization experiments, the cells were seeded into an 8-chambered cover glass with a density of 3 × 10^4^ cells/chamber and subsequently incubated at 37 °C in a humidified atmosphere containing 5% of CO_2_ for 24 h. After 24 h cells, the were washed three times with DPBS. The cells were incubated with Hoechst for 30 min at 37 °C. Afterwards, the cells were washed two times with DPBS and incubated with one of the red-emitting commercial probes. For mitochondria staining, the cells were treated with 200 nM MitoTracker Red FM for 40 min at 37 °C. For lysosome staining, the cells were incubated with 75 nM LysoTracker Deep Red for 120 min at 37 °C. For LD staining, the cells were incubated with 314 nM Nile Red for 10 min at room temperature and protected from exposure to light. Afterwards, the cells were washed two times with DPBS and stained with BDP-H diluted in DPBS for 60 min at 37 °C. After BDP-H staining, the cells were washed three times with DPBS.

For the BDP-H and Nile Red colocalization experiments, separate control samples with MCF-7 and MDA-MB-231 cells were prepared for finding the detection parameters that eliminate emission bleed-through from Nile Red in the green channel (501–590 nm) where the fluorescence of BDP-H was detected.

### 3.7. Cellular Microscopy

Breast cancer cells were imaged using a confocal Nikon Eclipse Te2000-U microscope (Nikon, Yokohama, Japan) with a C1si laser scanning confocal system. The microscope was equipped with a Fianium WhiteLase Micro supercontinuum laser (NKT Photonics, Birkerød, Denmark) with a pulse repetition rate of 30 MHz and filtered using 410 ± 5 nm (for Hoechst), 480 ± 5 nm (for BDP-H and BDP-NO_2_) and 560 ± 5 nm (for all red-emitting commercial probes) band-pass filters (all from Thorlabs Inc., Newton, NJ, USA). Imaging was performed using an 60×/1.4 NA oil immersion objective (Plan Apo VC, Nikon, Yokohama, Japan). The 32-channel spectral detector was applied to investigate the fluorescence of BDP-NO_2_ and BDP-H in live cells. The brightfield mode of Nikon Eclipse Te2000-U and the three-channel RGB detector with band-pass filters of 450/34 nm, 546/89 nm and 688/134 nm for blue, green and red channels, respectively, were used. Live cells were maintained at 37 °C in the Microscope Stage Incubation System (OkoLab, Pozzuoli, Italy) in a humidified atmosphere containing 5% of CO_2_ (0.80 Nl/min O_2_ and 0.04 Nl/min CO_2_). Image processing was performed using the EZ-C1 Bronze software (v.3.80, Nikon, Tokyo, Japan) and ImageJ software (v.1.53e, U.S. National Institutes of Health, Bethesda, MD, USA) [65].

### 3.8. Colocalization Analysis

Colocalization analysis of BDP-H and commercial probes (MitoTracker Red FM, LysoTracker Deep Red and Nile Red) was conducted using Fiji’s Coloc 2 plugin [66]. Average Pearson correlation coefficients ± standard deviation (SD) for each group were calculated from ten manually selected region of interests (ROI) each containing a single cell with the nuclear region excluded.

### 3.9. Fluorescence Lifetime Imaging Microscopy

Fluorescence lifetime imaging microscopy (FLIM) was performed using a Lifetime and Fluorescence Correlation Spectroscopy Upgrade for Nikon C1si (PicoQuant GmbH, Berlin, Germany) confocal laser scanning microscope. A Fianium WhiteLase Micro supercontinuum laser (NKT Photonics, Birkerød, Denmark) with a pulse repetition rate of 30 MHz and 480 ± 5 nm band-pass filter (Thorlabs Inc.) was used as an excitation source for BDP-H. The fluorescence lifetime signal of BDP-H was detected through a 578/105 nm filter (Thorlabs Inc.) using a single photon-counting avalanche photodiode (SPAD) (Micro Photon Devices, Bolzano, Italy). Detected photons were counted by a time-correlated single photon counter PicoHarp 300 (PicoQuant GmbH, Berlin, Germany). Each lifetime image was obtained by collecting 500 counts/pixel and the image resolution was 512 × 512 pixels. FLIM images were reconstructed using SymPhoTime 64 software (v.2.4.4874, PicoQuant GmbH, Berlin, Germany). A two-exponential reconvolution fitting model was used to determine the average fluorescence lifetime in each pixel of the FLIM image. The IRF applied for fitting was calculated by the SymPhoTime 64 software.

### 3.10. Statistical Analysis

The statistical analysis was conducted with rstatix (version 0.7.0) using R (version 4.1.2). The assumption of the normality of the data (microviscosity values of LDs from MCF-7 and MDA-MB-231 cells, two samples) was confirmed using a Shapiro-Wilk test. An *F*-test for the equality of two variances resulted in a rejection of the hypothesis that the two variances are equal. Therefore, Welch’s two-sample *t*-test was used to identify statistically significant differences between the mean microviscosity values of LDs from MCF-7 and MDA-MB-231 cells. A significance level of α = 0.05 was set. The results from Welch’s two-samples *t*-test are: t(20.34) = 6.88, *p* < 0.0001; the difference between means was 75 cP, 95% CI [53, 99], d* = 2.13, where CI—confidence interval, d*—Cohen’s *d* with Hedges’ correction. Data are representative of at least three experiments and is expressed in the following form: mean ± SD.

## 4. Conclusions

In summary, we highlight the importance of conducting a photophysical characterization of novel molecular rotors in aqueous media with or without proteins before their application in live biological models. Our work has led us to conclude that BDP-NO_2_ molecules form aggregates in aqueous media. Thus, the molecular rotor is unsuitable for biological systems. In contrast, the BDP-H molecular rotor exhibits better solubility in aqueous media and is more applicable for microviscosity measurements in live cells. Furthermore, our study delivers evidence that despite the interaction between BDP-H and protein molecules, the intracellular accumulation of the fluorophore is unaffected. It diffuses through the plasma membrane of both live and fixed human breast cancer cells and mainly accumulates in LDs. Furthermore, slight accumulation is visible in cytosol. Finally, we successfully utilize the BDP-H molecular rotor for the measurement of LD microviscosity in human breast cancer cells lines of different malignancy. For the first time, we demonstrate a significant difference between LD microviscosity in MCF-7 and MDA-MB-231 cancer cells. We anticipate that LD microviscosity will become a novel biomarker for the identification of the malignancy of cancer cells, and that the BDP-H molecular rotor could be used for this purpose.

## Data Availability

Raw data available upon reasonable request.

## Supplementary Materials

The following supporting information can be downloaded at: https://www.mdpi.com/article/10.3390/ijms23105687/s1.
