# Supplementary material for: Exploring BODIPY-Based Sensor for Imaging of Intracellular Microviscosity in Human Breast Cancer Cells
Source: Int J Mol Sci. 2022 May 19;23(10):5687. doi: 10.3390/ijms23105687 (PMC9143602; doi:10.3390/ijms23105687)
Supplement: Supplementary file 1 [file ijms-23-05687-s001.zip › ijms-1698052-supplementary.pdf]

International Journal of *Molecular Sciences*

MDPI

## Supplementary Material

# Exploring BODIPY-Based Sensor for Imaging of Intracellular Microviscosity in Human Breast Cancer Cells

**Dziugas Jurgutis [1,2], Greta Jarockyte [1], Vilius Poderys [1], Jelena Dodonova-Vaitkuniene [3], Sigitas Tumkevicius [3], Aurimas Vysniauskas [2], Ricardas Rotomskis [1] and Vitalijus Karabanovas [1,4,*]**

1 Biomedical Physics Laboratory, National Cancer Institute, P. Baublio st. 3b, 08406 Vilnius, Lithuania; dziugas.jurgutis@nvi.lt (D.J.); greta.jarockyte@nvi.lt (G.J.); vilius.poderys@nvi.lt (V.P.); ricardas.rotomskis@nvi.lt (R.R.)
2 State Research Institute Center for Physical Sciences and Technology, Sauletekio Ave. 3, 10257 Vilnius, Lithuania; aurimas.vysniauskas@ftmc.lt
3 Institute of Chemistry, Faculty of Chemistry and Geosciences, Vilnius University, Naugarduko St. 24, 03225 Vilnius, Lithuania; jelena.dodonova@gmail.com (J.D.-V.); sigitas.tumkevicius@chf.vu.lt (S.T.)
4 Department of Chemistry and Bioengineering, Vilnius Gediminas Technical University, Sauletekio Ave. 11, 10223 Vilnius, Lithuania
* Correspondence: vitalijus.karabanovas@nvi.lt

## 1. Normalized absorbance and fluorescence spectra

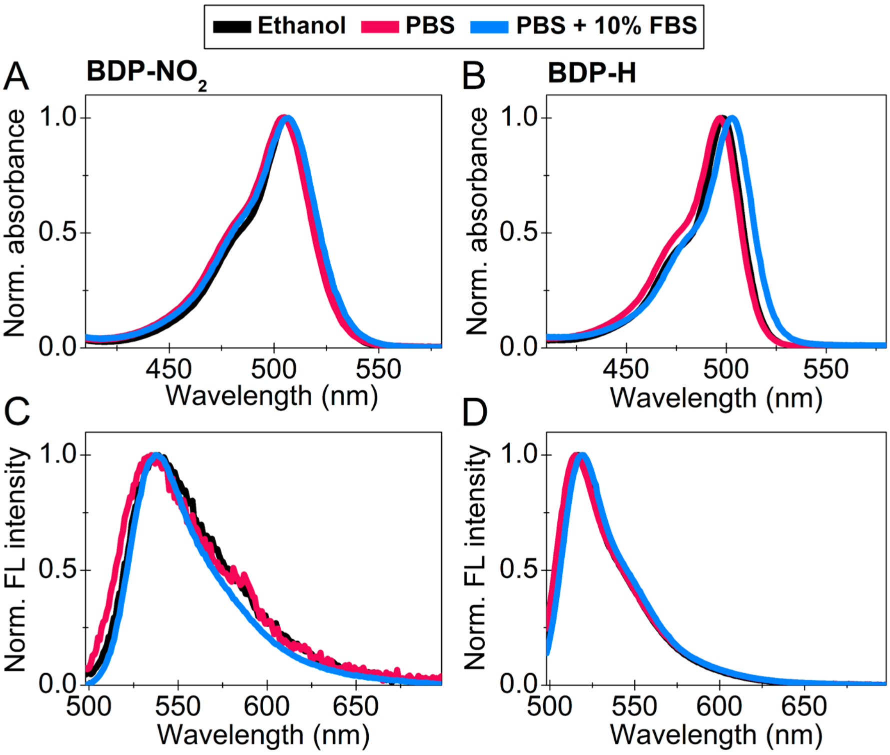


**Figure S1.** Normalized (Norm.) spectra of BDP-$NO_2$ and BDP-H diluted in ethanol (black), 7.4 pH phosphate-buffered saline (PBS) (red) or PBS with 10% fetal bovine serum (FBS) (blue). **A**, **B** – norm. absorption spectra. **C**, **D** – norm. fluorescence (FL) spectra, $\lambda_{ex}$ = 488 nm.

## 2. Fitting parameters of fluorescence decays from the molecular rotors in aqueous media

**Table S1.** Fitting parameters of BDP-$NO_2$ and BDP-H fluorescence decays in ethanol and aqueous media (Figure 2E, F). Amplitudes are scaled by the integrated instrument response function. Concentration of both dyes was 9 µM.

| Sample | Exponent component | $\alpha$ [1] | $\tau$ (ps) | $f$ [2] (%) | $\tau_{Av}$ [3] (ps) | $\chi^2$ |
|---|---|---|---|---|---|---|
| BDP-$NO_2$ in ethanol | 1 ($\tau_1$) | 0.2300 | 64 | 100 | 64 | 1.13 |
| BDP-$NO_2$ in PBS | 1 ($\tau_1$)<br>2 ($\tau_2$) | 0.2541<br>0.0003 | 57<br>1958 | 96.69<br>3.31 | 131 | 1.07 |
| BDP-$NO_2$ in PBS with 10% FBS | 1 ($\tau_1$)<br>2 ($\tau_2$)<br>3 ($\tau_3$) | 0.0186<br>0.0094<br>0.0087 | 610<br>2822<br>7392 | 11.11<br>25.95<br>62.94 | 5453 | 1.08 |
| BDP-H in ethanol | 1 ($\tau_1$) | 0.0709 | 261 | 100 | 261 | 1.15 |
| BDP-H in PBS | 1 ($\tau_1$) | 0.0701 | 264 | 100 | 264 | 1.01 |
| BDP-H in PBS with 10% FBS | 1 ($\tau_1$)<br>2 ($\tau_2$)<br>3 ($\tau_3$) | 0.0280<br>0.0104<br>0.0071 | 364<br>2379<br>7000 | 12.08<br>29.24<br>58.68 | 4850 | 1.12 |

[1] Amplitude or pre-exponential factor.
[2] Fraction of fluorescence intensity (scales with the amplitude and the lifetime).
[3] Intensity-weighted mean lifetime.

## 3. Gradual decrease in BDP-$NO_2$ absorbance in aqueous media

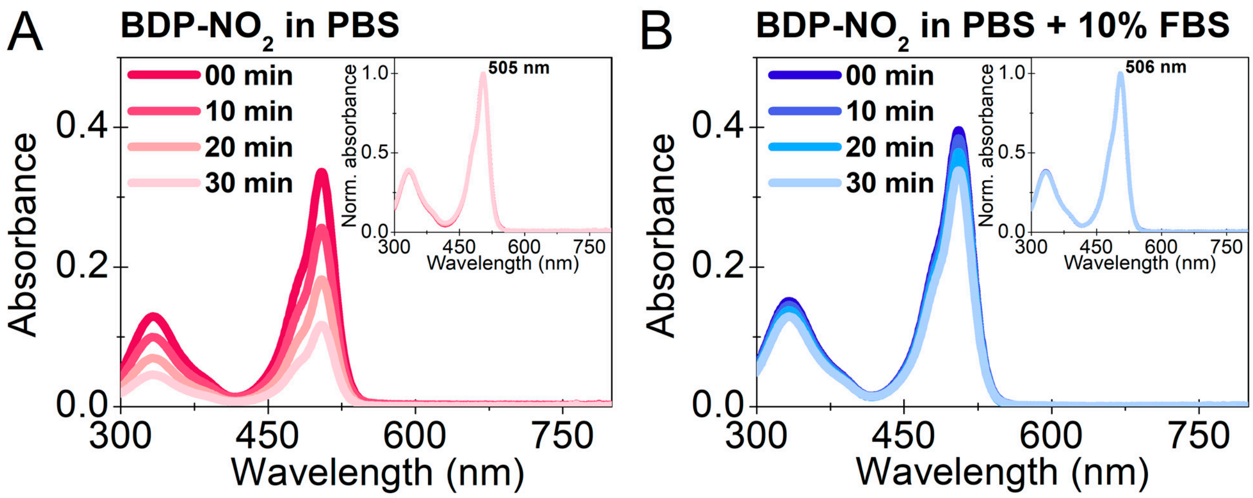


**Figure S2.** Absorption spectra of BDP-$NO_2$ diluted in PBS (**A**) and PBS + 10% FBS (**B**). Insets show normalized (norm.) spectra. BDP-$NO_2$ absorbance dwindles with time (65.23% decrease in 30 min) indicating that the optical properties of the molecule are not stable in aqueous media. Addition of FBS slightly improves stability of BDP-$NO_2$, however the decrease in absorbance with time was still observed, although at a much slower rate (14.58% decrease in 30 min).

## 4. Photostability of BDP-H in PBS

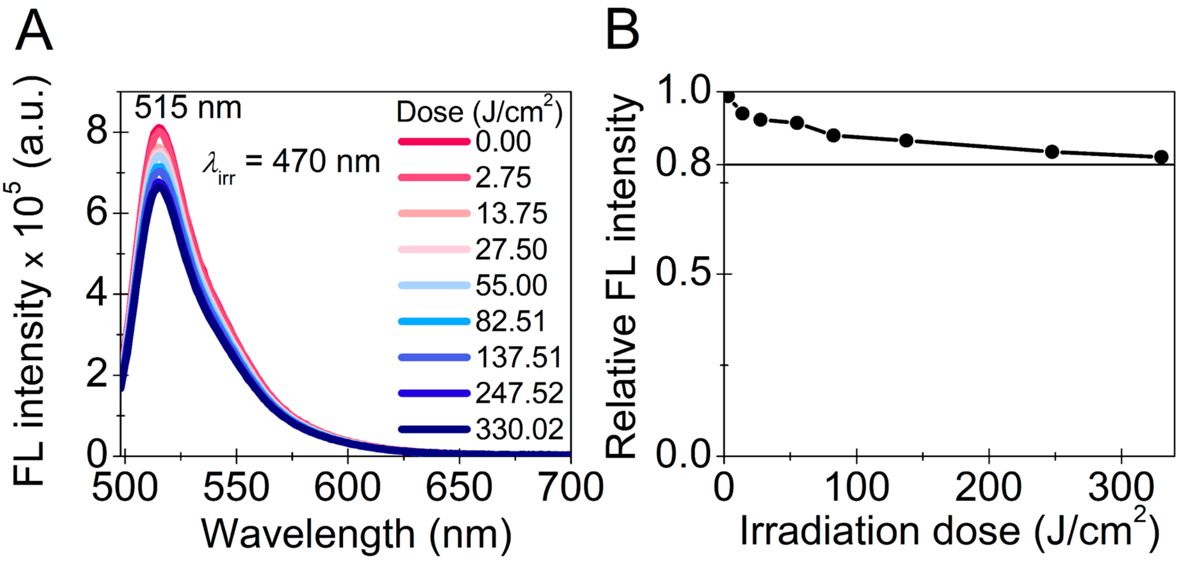


**Figure S3.** Photostability of BDP-H in PBS. **A** – fluorescence (FL) spectra of 9 μM BDP-H in PBS after irradiation with 470 nm light (laser power – 36 mW). FL spectra were obtained under the excitation at 488 nm. **B** – BDP-H photobleaching curve. Values of relative FL intensity were obtained by dividing the maximum FL spectrum value (at 515 nm) of non-irradiated sample from the maximum FL values after each irradiation.

## 5. Photophysical characterization of BDP-H interaction with BSA

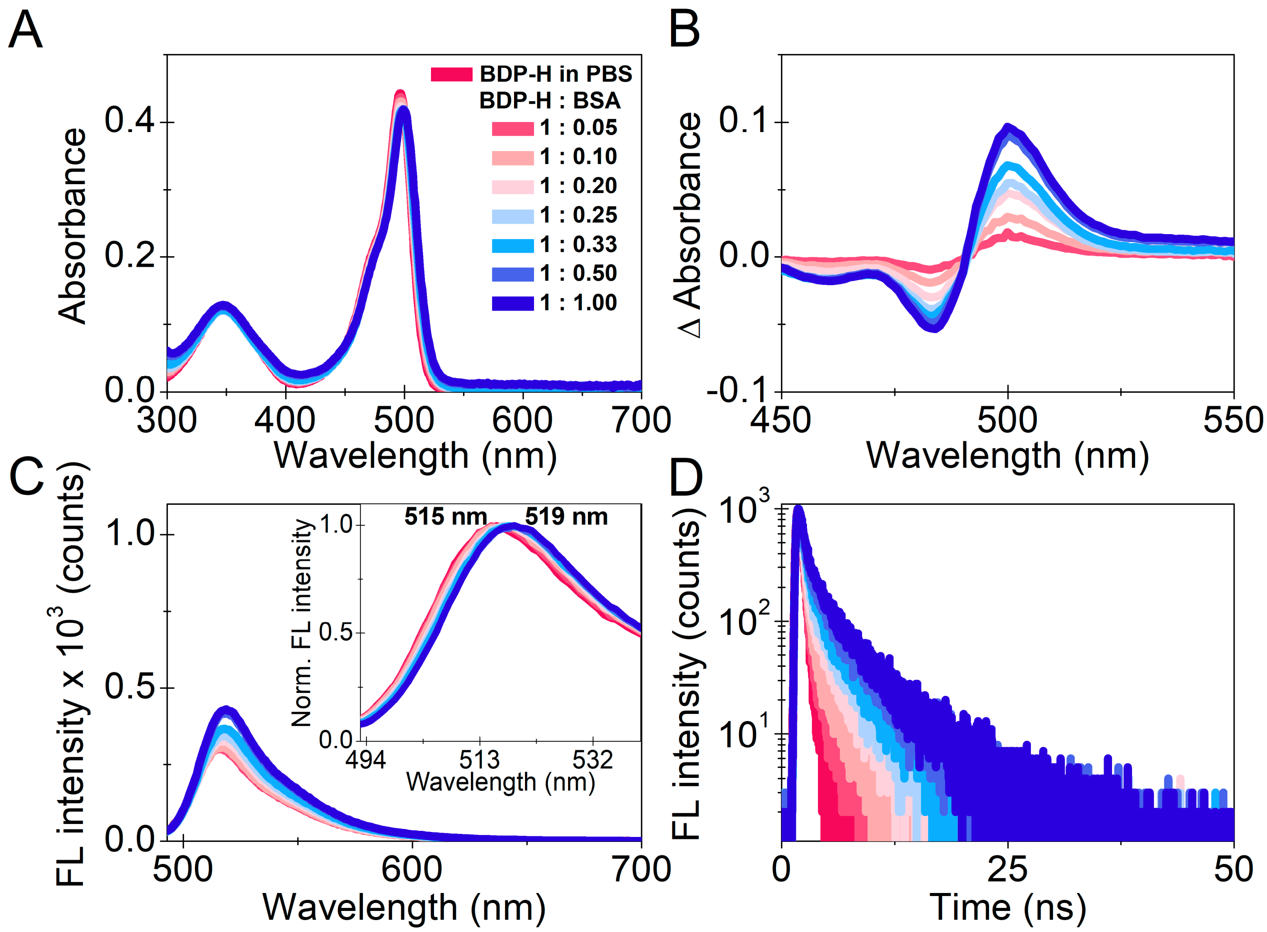


**Figure S4.** Photophysical characterization of 9 μM BDP-H in PBS with increasing concentrations of bovine serum albumin (BSA) (up to 9 μM) measured in PBS. The color legend of BDP-H and BSA ratio is shown in **A** panel. The absorption of BDP-H in PBS was subtracted from each BDP-H + BSA spectra (**A**), which resulted in **B**. The changes of fluorescence emission are represented in **C**, with the inset showing normalized fluorescence spectra. **D** – comparison of time-resolved fluorescence decays, $\lambda_{ex}$ = 473 nm.

## 6. BDP-H staining in live and fixed human breast cancer cells

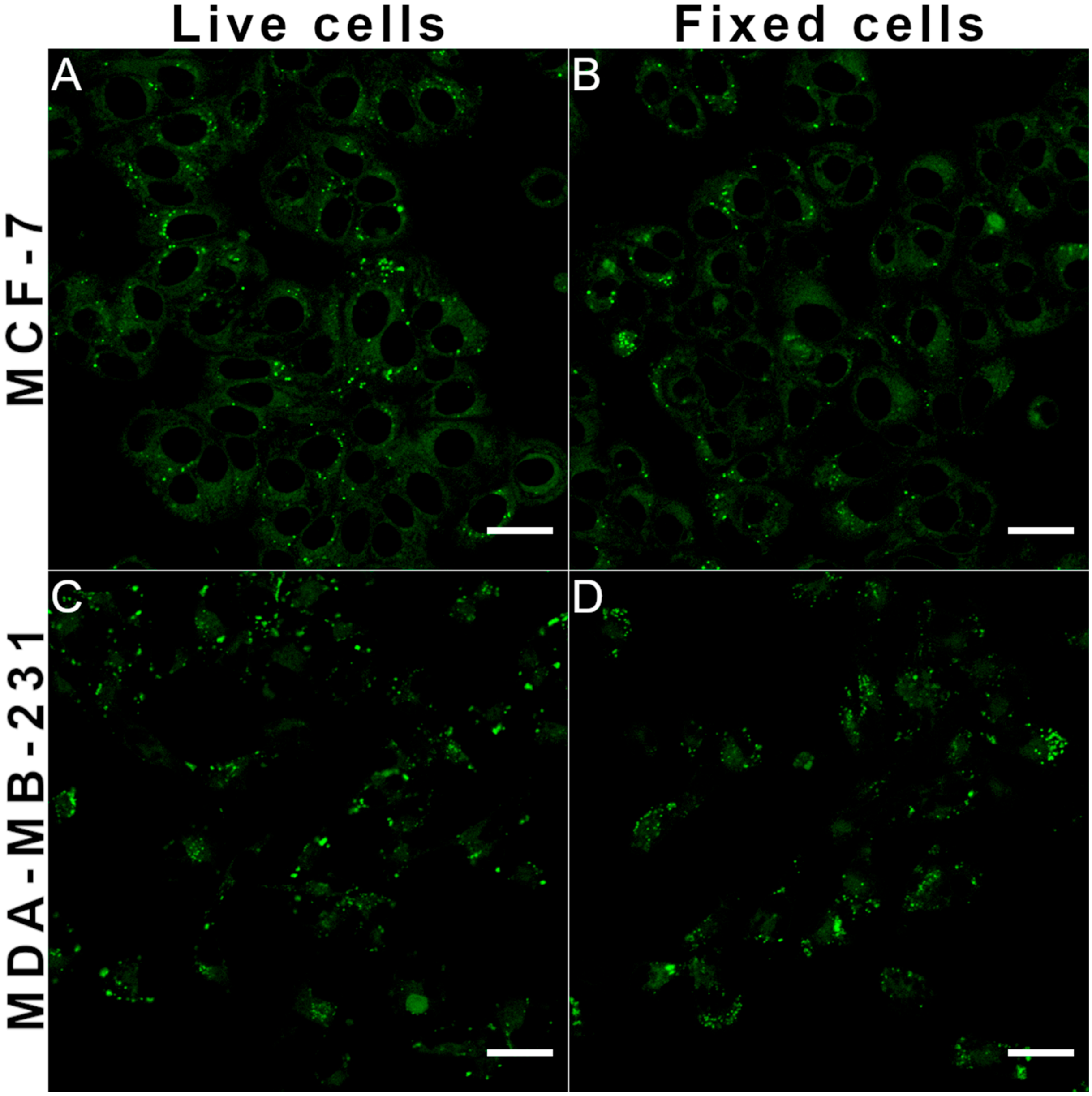


**Figure S5.** Comparison of BDP-H staining in live and fixed MCF-7 and MDA-MB-231 human breast cancer cells. Live cells (**A**, **C**) were incubated with 9 μM BDP-H (diluted in PBS) for 60 min. Cells in **B** and **D** were fixed using 4% paraformaldehyde (PFA) and afterwards stained with 9 μM BDP-H (diluted in PBS) for 60 min. Cells were visualized using laser-scanning confocal microscope; $\lambda_{ex}$ = 480±5 nm. Scale bars: 30 μm.

## 7. BDP-H colocalization analysis

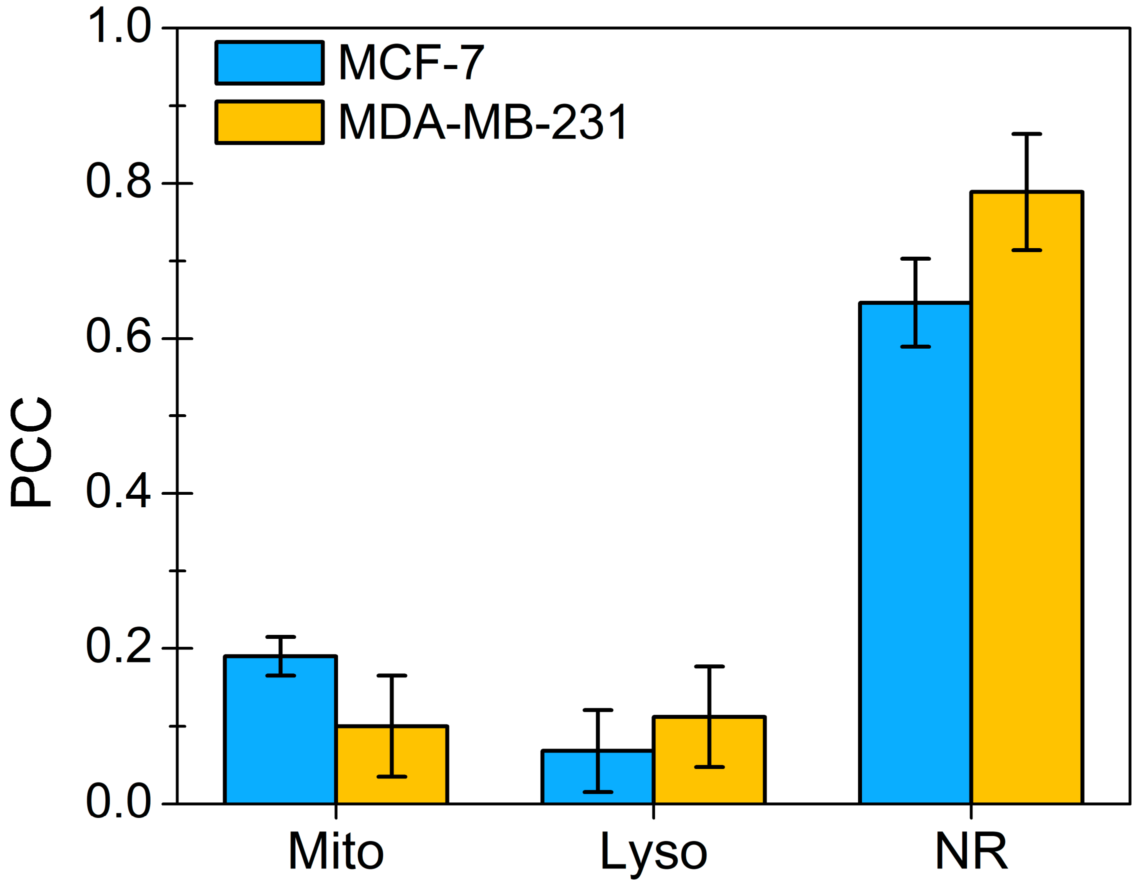


**Figure S6.** Pearson correlation coefficients (PCC) obtained from colocalization analysis of BDP-H and the following commercial probes: MitoTracker Red FM (Mito), LysoTracker Deep Red (Lyso), Nile Red (NR) in MCF-7 and MDA-MB-231 cells. The average PCC ± SD were calculated from ten ROI, each containing a single cell with the nuclear region excluded. Fiji's Coloc 2 plugin was used to obtain the PCC values.

## 8. Fluorescence and FLIM images of unstained human breast cancer cells

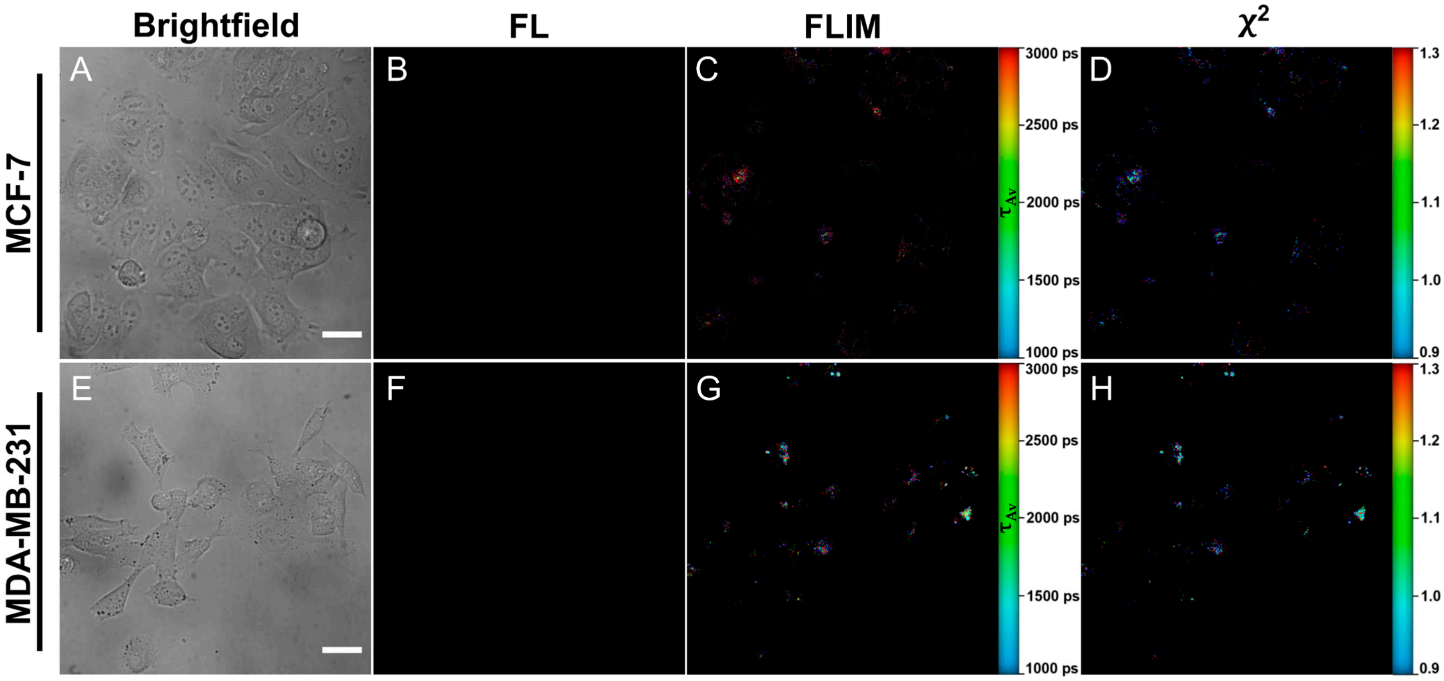


**Figure S7.** Confocal and fluorescence lifetime imaging microscopy (FLIM) images of unstained human breast cancer cells. **A**, **E** – brightfield images; scale bars: 30 μm. Autofluorescence of cells is not visible in fluorescence (FL) images (**B**, **F**) since imaging parameters were chosen specifically to nullify it. However, weak autofluorescence signal is evident in FLIM images (**C**, **G**); the fluorescence was excited at 480±5 nm and detected through a 578/105 nm filter. **D**, **H** – $\chi^2$ images obtained from the analysis of the FLIM images. Fitting results of the aforementioned FLIM images are shown in Figure S8 and Table S2.

## 9. Fluorescence decays and residuals obtained from FLIM images of unstained cells

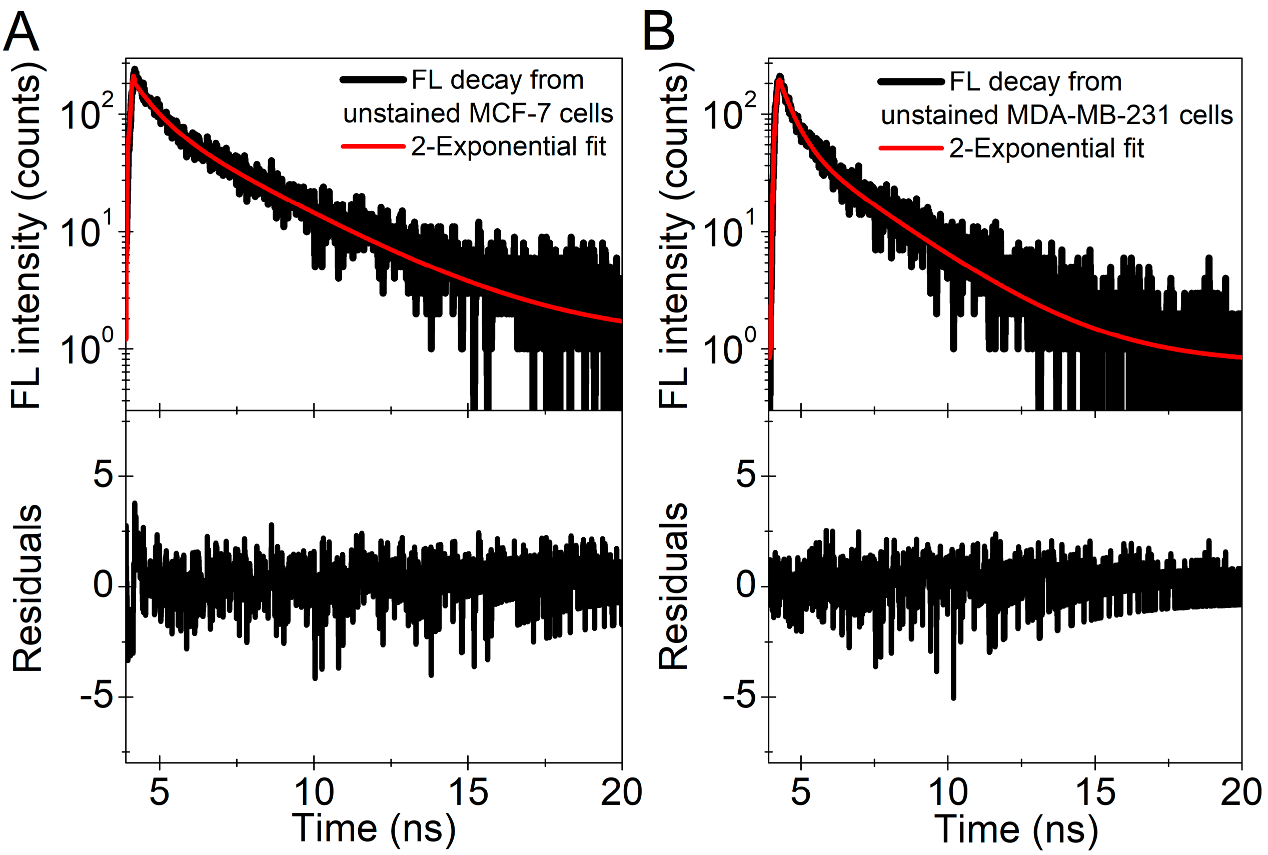


**Figure S8.** Fluorescence decays (black) with biexponential reconvolution fits (red line) obtained from FLIM images of unstained MCF-7 and MDA-MB-231 cells (Figure S7C, G). Residuals (difference between the fitted decay curve and raw data) are shown below the decay curves.

## 10. Fitting parameters of fluorescence decays obtained from FLIM images of unstained cells

**Table S2.** Fluorescence lifetimes, amplitudes and other fitting parameters of fluorescence decays obtained from FLIM images of unstained MCF-7 and MDA-MB-231 cells displayed in Figure S7.

| Cell line (FLIM image) | Exponent component | $\tau$ (ps) | Amplitude (kCnts) | $\tau_{Av}$ [1] (ps) | $\chi^2$ |
|---|---|---|---|---|---|
| MCF-7 (Figure S6C) | 1 ($\tau_1$) | 570 | 0.12 (57.14%) | 2420 | 1.10 |
| | 2 ($\tau_2$) | 2920 | 0.09 (42.86%) | | |
| MDA-MB-231 (Figure S6G) | 1 ($\tau_1$) | 522 | 0.18 (78.26%) | 1990 | 1.00 |
| | 2 ($\tau_2$) | 2900 | 0.05(21.74%) | | |

[1] Intensity-weighted mean lifetime.

## 11. BDP-H calibration curve

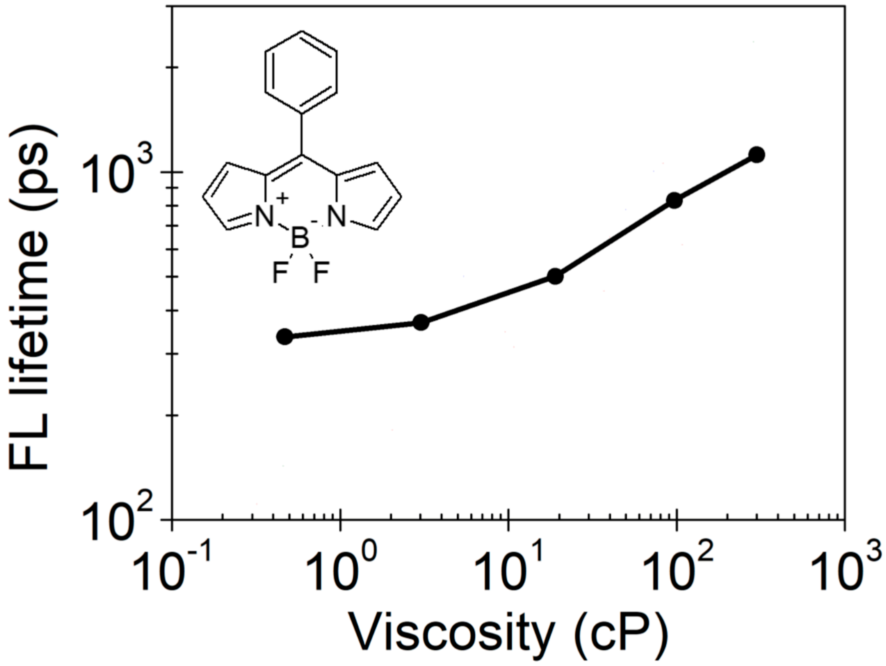


**Figure S9.** BDP-H calibration curve obtained at 40 °C in toluene-castor oil mixtures [1].

## 12. Average values of fitting parameters obtained from FLIM images of cancer cells stained with BDP-H

**Table S3.** Average values of fitting parameters of fluorescence decays obtained from the analysis of 40 FLIM images (20 FLIM images for each cell line).

| Cell line | Exponent component | $\tau_{1,2\_Av}$ [1] (ps) | Average amplitude (kCnts) | $\tau_{Av}$ [2] (ps) | $\chi^2_{Av}$ | Microviscosity [3] (cP) |
|---|---|---|---|---|---|---|
| MCF-7 | 1 ($\tau_{1\_Av}$)<br>2 ($\tau_{2\_Av}$) | 875±18<br>3675±23 | 1.78±2.17<br>0.04±0.05 | 1158±53 | 1.06±0.05 | 119.69±8.51 |
| MDA-MB-231 | 1 ($\tau_{1\_Av}$)<br>2 ($\tau_{2\_Av}$) | 992±64<br>2862±396 | 1.08±2.06<br>0.16±0.30 | 1436±194 | 1.03±0.06 | 195.33±48.30 |

[1] Average lifetime of $\tau_1$ or $\tau_2$ components.

[2] Average intensity-weighted mean lifetime.

[3] Microviscosity calculated from $\tau_{1\_Av}$ using the BDP-H calibration graph in Figure S9.

## 13. Fluorescence and FLIM images of human breast cancer cells stained with BDP-H

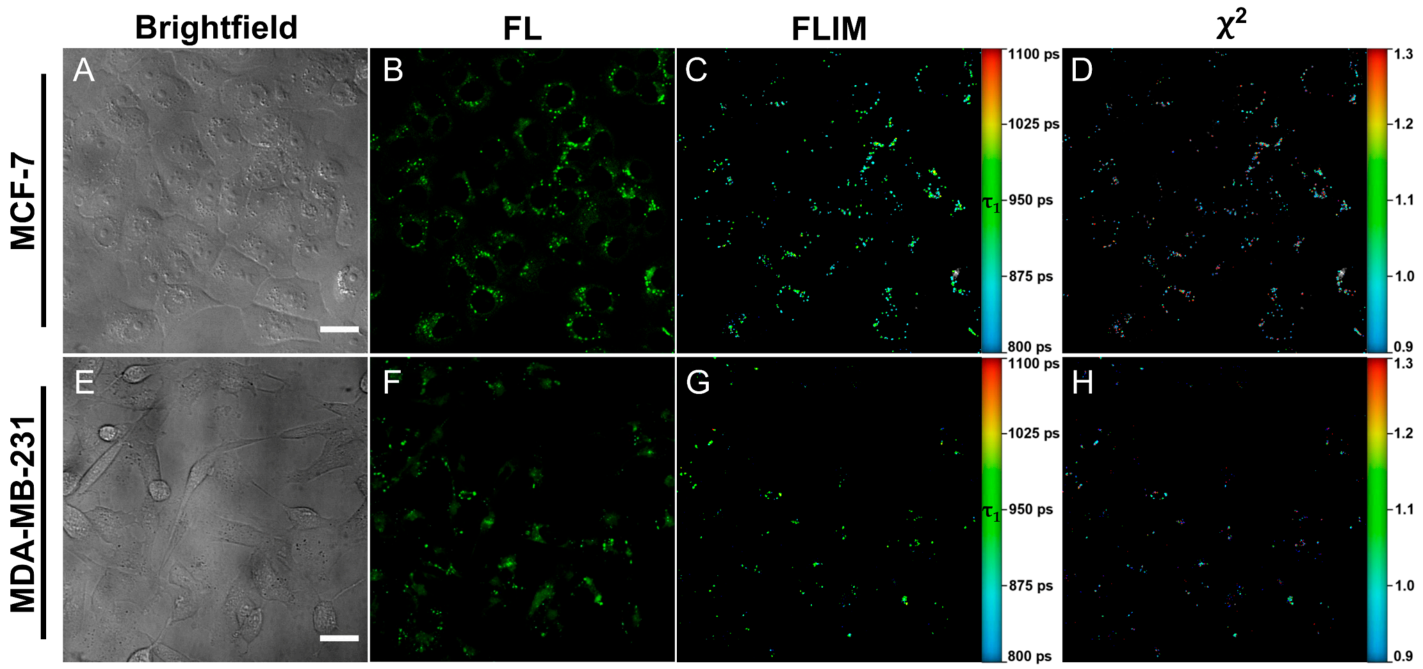


**Figure S10.** Confocal and FLIM images of human breast cancer cells stained with BDP-H molecular rotor. **A**, **E** – brightfield images; scale bars: 30 μm. **B**, **F** – fluorescence (FL) images. **C**, **G** – FLIM images. The fluorescence was excited at 480±5 nm and detected through a 578/105 nm filter. **D**, **H** – $\chi^2$ images obtained from the analysis of the FLIM images. Fitting results of the aforementioned FLIM images are shown in Figure S11 and Table S4.

## 14. Fluorescence decays and residuals obtained from FLIM images of human breast cancer cells stained with BDP-H

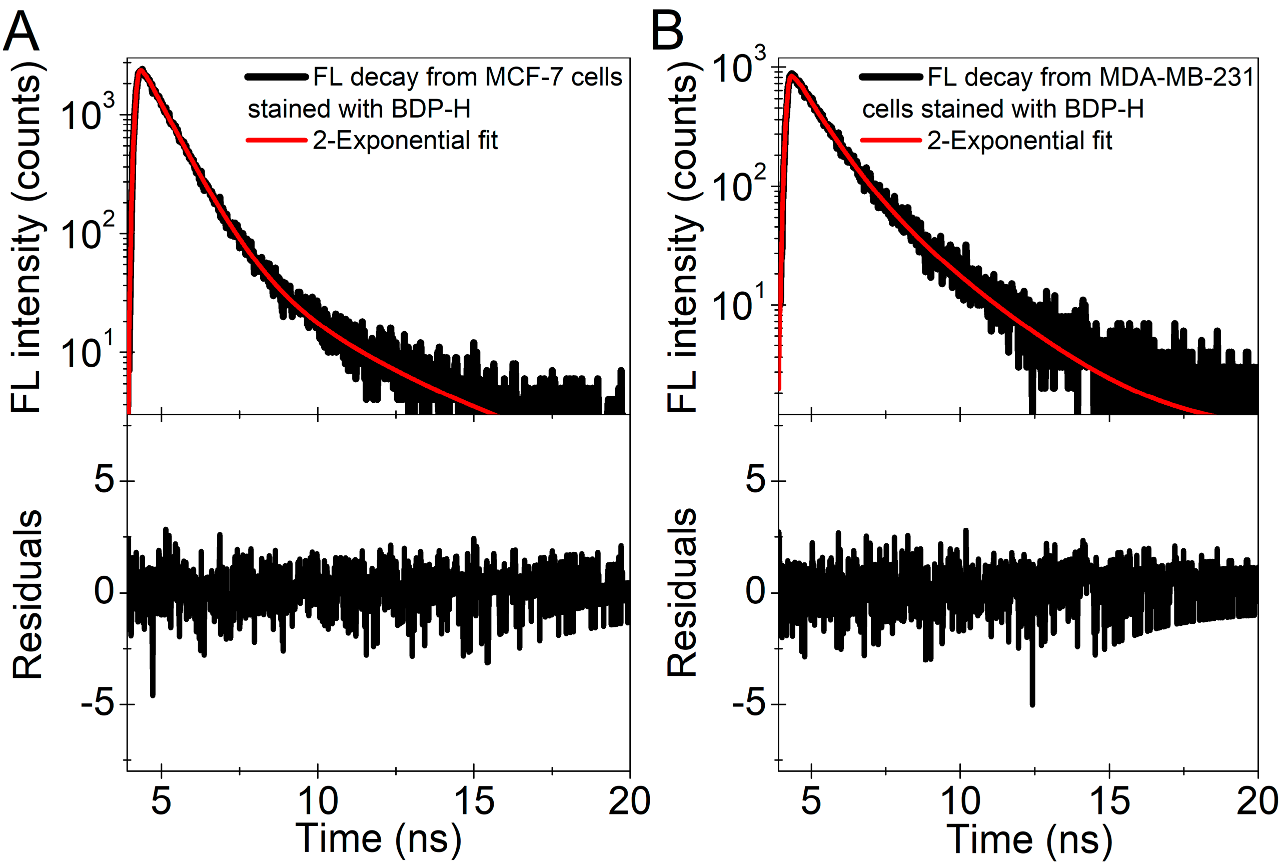


**Figure S11.** Fluorescence decays (black) with biexponential reconvolution fits (red line) obtained from FLIM images of MCF-7 and MDA-MB-231 cells stained with BDP-H (Figure 6B, D or Figure S10C, G). Residuals (difference between the fitted decay curve and raw data) are shown below the decay curves.

## 15. Fitting parameters of fluorescence decays obtained from FLIM images of human breast cancer cells stained with BDP-H

**Table S4.** Fluorescence lifetimes, amplitudes and other fitting parameters of fluorescence decays obtained from FLIM images displayed in Figure 6B, D or Figure S10C, G.

| Cell line (FLIM image) | Exponent component | $\tau$ (ps) | Amplitude (kCnts) | $\tau_{Av}$ [1] (ps) | $\chi^2$ |
|---|---|---|---|---|---|
| MCF-7 (Figure 6B) | 1 ($\tau_1$) | 836 | 3.04 (97.43%) | 1080 | 0.98 |
| | 2 ($\tau_2$) | 3300 | 0.08 (2.56%) | | |
| MDA-MB-231 (Figure 6D) | 1 ($\tau_1$) | 927 | 0.93 (83.78%) | 1410 | 1.08 |
| | 2 ($\tau_2$) | 2400 | 1.18 (16.22%) | | |

[1] Intensity-weighted mean lifetime.

# Reference

1. Toliautas, S.; Dodonova, J.; Žvirblis, A.; Čiplys, I.; Polita, A.; Devižis, A.; Tumkevičius, S.; Šulskus, J.; Vyšniauskas, A. Enhancing the Viscosity-Sensitive Range of a BODIPY Molecular Rotor by Two Orders of Magnitude. *Chemistry – A European Journal* **2019**, *25*, 10342–10349, doi:10.1002/chem.201901315.
